# Psychosocial Impact of Electrical Burn in Children: A Follow-Up Study Conducted at a Tertiary Care Hospital

**DOI:** 10.7759/cureus.32816

**Published:** 2022-12-22

**Authors:** Faizan Shahid, Mudassar Fiaz Gondal, Noor Us Sabah, Roohmah Chaudhry, Hasnain Aslam, Usama Iftikhar, Omer Fraz, Sajeel Saeed, Jawad Basit

**Affiliations:** 1 Department of Pediatric Surgery, Holy Family Hospital, Rawalpindi, PAK; 2 Department of Surgery, Jinnah Hospital, Lahore, PAK; 3 Department of Surgery, Rawalpindi Medical University, Rawalpindi, PAK; 4 Department of Medicine, Rawalpindi Medical University, Rawalpindi, PAK

**Keywords:** burn injury, traumatic amputation, body dysmorphic disorder, electric burn, psychosocial impact

## Abstract

Introduction

Electrical burn injuries are very common in the pediatric population and are usually accidental and sometimes occupational. The objective of our study was to evaluate the epidemiology of electrical burn injuries and prospectively evaluate the long-term psychosocial impact of electrical burn injuries in children.

Materials and methods

A qualitative interview study was conducted prospectively among sixty patients presented to Holy Family Hospital, Rawalpindi, Pakistan. Demographic details, mode of presentation, detail of injury, total body surface area, initial condition, and surgical interventions were noted during their stay at the hospital. Their physical and psychological outcomes were evaluated by administering the Strength and Difficulties Questionnaire (SDQ) and Body Dysmorphic Disorder Questionnaire (BDDQ) via telephonic interviews after six weeks of discharge from the hospital. The study was conducted over a span of 1 year from January 2021 to January 2022.

Results

There were 60 patients who presented to the department of pediatric surgery during the span of the study. The mean age was 9.9 years ± 3.133 years (SD) and 80% of the patients were above 8 years of age with a male-to-female percentage of 86.67% to 13.33%, respectively. Forty-two (70%) patients incurred high-voltage electrical burns while 18 (30%) suffered low-voltage electrical burns. Mortality was 13.33% (n=8). Out of 52 patients who survived, 35 (67.30%) were labeled as having Body Dysmorphic Disorder upon administration of BDDQ through a six-week follow-up. The majority of the patients had abnormal or borderline results in different scales of SDQ.

Conclusion

The long-term psychological stress and the varied spectrum of psychiatric disorders in electrical burn patients are profound. The prevention of burn injuries can be effectively achieved by educating parents about safety measures and improving health infrastructure. Implementation of a dedicated national program for psychological support of burn patients should be made accessible to all patients.

## Introduction

Burn has been declared a preventable global health problem according to WHO's latest census. While the mortality associated with burns is reaching an alarming high of 180,000 deaths per year, the more concerning aspect is that the majority of these burns occur in lower and middle socioeconomic countries, especially in African and South Asian regions. In Bangladesh, Colombia, Egypt, and Pakistan, 17% of the children with burns have a temporary disability and 18% have a permanent disability [1]

Burn of all sorts and its effective management proposes a grave challenge for emergency physicians as well as surgeons everywhere across the globe. According to the latest statistics, among all the types of burns, pediatric burn constitutes the largest single group admitted and treated at any tertiary care unit in developed as well as developing countries. Pediatric burns can further be classified into two subgroups that are non-electrical and electrical burns [2] Electrical burns constitute up to 4-5% of all burn injuries according to the Advanced Trauma Life Support Manual [3]

Electrical burn injury (EBI) constitutes low-voltage and high-voltage injuries. Low- and high-voltage injuries constitute the injuries incurred by sources less and more than 1000V, respectively. High-voltage electrical burns are associated with poor surgical, medical and psychological outcomes and higher mortality compared to low-voltage electrical burns [4] Low voltage current is known to impact mostly young children at home while older children tend to get electrocuted more by high voltage current, mostly while playing [5]

Although much data has been published around the globe regarding the spectrum of injuries caused by electric burns and the economic and disability strain it puts on caregivers and patients, not many studies have been conducted on the psychosocial impact of this preventable health problem. Moreover, in clinical set-ups of developing countries, the psychosocial impact is the least concerning aspect of any illness.

The objective of our study was to prospectively evaluate electrical burn patients who were being managed in the ward of pediatric surgery at Holy Family Hospital (Rawalpindi, Pakistan) to establish the common mode of injuries, timing of presentation, and different management strategies.

## Materials and methods

After approval from the Ethical Review Board of Rawalpindi Medical University, we conducted a descriptive cross-sectional study of all the pediatric patients of electric burns presenting to the department of pediatric surgery, Holy Family Hospital. Data was taken regarding their demographic details, mode of presentation, detail of injury, Total body surface area, initial condition, surgical intervention, and their outcome during the period of **one **year from January 2021 to January 2022.

To evaluate the psychosocial impact, a questionnaire was devised with three components: demographic details, the Strength and Difficulties Questionnaire, and the Body Dysmorphic Disorder Questionnaire. Data regarding body dysmorphia and overall strengths and difficulties was then carried out via telephonic conversation with the patient and their primary caregiver after six weeks of discharge from the hospital.

Strength and Difficulties Questionnaire

The Strengths and Difficulties Questionnaire (SDQ) is a brief measure of psychological adjustment and psychopathology of 3- to 16-year-olds and can be completed by children, adolescents, parents, and teachers. The SDQ is sensitive to change over a short period of time and is a widely used instrument in research related to mental health. Though not a diagnostic tool, SDQ efficiently measures in which areas of life, a child is facing difficulty and also appropriately gauges the impact of any such difficulty in everyday life [6]

The SDQ covers 25 attributes grouped into five subscales: 1) emotional symptoms (for example “I am often unhappy, downhearted, or tearful”), 2) conduct problems (for example, “I fight a lot”), 3) hyperactivity problems (for example, “I think before I do things”), 4) peer problems (for example, “I have one good friend or more”), and 5) prosocial behavior (for example “I try to be nice to other people”) [7]

Psychometric properties of the SDQ have been assessed in a representative sample of 10,000 children aged 5 to 15 years, all of whom also had psychiatric assessments. Findings confirmed and extended previous reports of satisfactory reliability and validity. This measure was used for the entire age sample in this study (8-17 years) as Maskell et al inferred that children as young as 7 years can report as reliably as older children. The SDQ parent report has been examined in terms of the psychometric properties within an Australian pediatric population and has demonstrated positive use as a measure of psychopathology in young Australian children [8] The sensitivity and specificity of SDQ to diagnose psychiatric disorders have been found out to be 85% and 80%, respectively [9]

Body Dysmorphic Disorder Questionnaire

The Body Dysmorphic Disorder Questionnaire (BDDQ) is a validated tool to study the effect of an imagined body defect that causes a negative effect on functioning [10]. It also asks about the concerns related to physical appearance as well as how much time a person spends thinking about themself and how they see themself fit within society.​​​​ With a sensitivity of 94%, a specificity of 90%, and a likelihood ratio of 9.4, the BDDQ has proved to have good concurrent validity [11].

## Results

There were 60 patients presented to our department of pediatric surgery, Holy Family Hospital, Rawalpindi. Among these, the mean age was found to be 9.9 years ± 3.133 years (SD), and 48 patients (80%) were above 8 years of age, with a male predominance of 86.67% (n=52) and a female percentage of 13.33% (n=8). Table 1 shows the demographic details.

**Table 1:** Frequency and percentage of demographic variables

Demographic Variable	n (%)
Gender	
Male	52 (86.67%)
Female	8 (13.33%)
Age groups	
Less than 5 years	4 (6.66%)
5-8 years	8 (13.33%)
8-11 years	31 (51.66%)
Greater than 11 years	17 (28.33%)

The total body surface area (TBSA) involved was categorized into five sub-groups: 0 was < 10%, 1 was 10-20-%, 2 was 20-30%, 3 was 30-40%, and 4 was >40%. 20% of the patients had TBSA <10% while the majority of the patients (51.61%) had a TBSA involvement of 10-20% (Table 2). 22 (70.96%%) patients had incurred high voltage electric burn injuries while 29.04% had an association with low voltage current (Table 2).

**Table 2:** Frequencies and percentages of total body surface area (TBSA) and mode of injury

Percent of TBSA affected	n (%)
<10%	12 (20%)
10-20%	31 (51.6%)
20-30%	6 (10%)
30-40%	4 (6.67%)
>40%	7 (11.6%)
Mode of Injury	
High voltage electrical burn	42 (70%)
Low voltage electrical burn	18 (30%)

There were unfavorable outcomes in 29 of our patients (48.33%) and the mortality rate was 13.33% (n=8), out of which one of the patients was received expired. 26 (43.33%) patients underwent amputations while 14 (23.33%) patients underwent fasciotomy and 14 (23.33%) underwent wound debridement. One patient had to undergo a suprapubic cyst-ostomy due to a severe burn of the perineal region while one (3.22%) patient underwent a diversion colostomy due to a deep abdominal wound perforating the gut viscus. The details are shown in Table 3. Only 11 patients out of 26 patients who had amputations were able to afford prosthetics; the others were not able to access these facilities due to financial constraints (Table 3). The most common mode of injury was by touching the wire close to the roof for chasing kites. None of the patients had guardian supervision when the injury resulted.

**Table 3:** The percentage of mortality in the initial cohort and frequencies and percentages of surgical interventions performed on the survivors

Variable	n (%)
Mortality	8 (13.33%)
Unfavorable outcomes	29 (48.33%)
Amputations	26 (43.33%)
Fasciotomy	14 (23.33%)
Prosthetics indicated in	26 (50.0%)
Prosthetics afforded by	11 (42.30%)
Could not afford prosthetics	15 (57.69%)
Wound debridement	14 (23.33%)
Suprapubic cyst-ostomy	2 (3.33%)
Diversion colostomy	1 (1.66%)

Fifty-two survivors were included in the evaluation of the psychosocial impact on our patients. Interviews were conducted via telephonic calls after six weeks of discharge from the hospital and BDDQ and SDQ were administered. 35 (67.30%) out of 52 patients who responded to our telephonic interviews were labeled as having body dysmorphic disorder and were further referred to a proper psychologist for evaluation and treatment.

The Strength and Difficulties Questionnaire was quite diverse in assessing the different aspects of the psychosocial health of our subjects. It was administered on parents as well as their children in order to assess the patients for the following five scales: Emotional Problem Scale, Conduct Problem Scale, Hyperactivity Scale, Peer Problem Scale, Prosocial Scale, and Total Difficulties Scale.

Nineteen (36.53%) out of 52 patients were labeled as having problems dealing with and expressing their emotions (Emotional Problem Scale, Table 4). 24 (46.15%) out of 52 patients had problems interacting with their peers, parents, and siblings as well caregivers and teachers (Conduct Problem Scale, Table 4). 10 patients (19.23%) fell in the borderline category for hyperactivity scale (Hyperactivity Scale, Table 4). 32 (61.53%) out of 52 had borderline disturbances when it came to social interaction with siblings and friends, while eight patients (15.38%) had abnormal behavioral patterns which needed proper treatment (Peer Problem Scale, Table 4). 36 patients (69.23%) out of 52 patients showed extreme frustration over being bullied and being name-called by their peers at school or in the neighborhood (Prosocial Scale, Table 4). 22 patients (42.30%) out of 52 had borderline disturbances when it came to social interaction with siblings and friends, while 10 (19.23%) patients had abnormal behavioral patterns which needed proper treatment (Total Difficulties Score, Table 4)

**Table 4:** Results of the administration of the Body Dysmorphic Questionnaire and the Strengths and Difficulties Questionnaire

Body Dysmorphic Disorder	n (%)
Yes	35 (67.30%)
No	17 (32.69%)
Strength and Difficulties Questionnaire	
Emotional Problem Scale	
Normal	33 (63.46%)
Borderline	6 (11.53%)
Abnormal	13 (25.00%)
Conduct Problem Scale	
Normal	28 (55.55%)
Borderline	8 (14.81%)
Abnormal	16 (29.62%)
Hyperactivity Scale	
Normal	42 (80.76%)
Borderline	10 (19.23%)
Abnormal	0 (00.00%)
Peer Problem Scale	
Normal	12 (23.07%)
Borderline	32 (61.53%)
Abnormal	8 (15.38%)
Prosocial Scale	
Normal	16 (30.76%)
Borderline	12 (23.07%)
Abnormal	24 (46.15%)
Total Difficulties Score	
Normal	20 (38.46%)
Borderline	22 (42.30%)
Abnormal	10 (19.23%)

## Discussion

Electrical burn injuries are comparatively rare, comprising 4-9% of all burn injuries [3] The main difference in electric burn injuries, among others, is that the extent of damage is much deeper and more devastating than the visible injury. As far as the mechanism of injury is concerned, these result from electrical shock generated depending on the voltage, amperage, tissue resistance, and duration of contact. The dryness of the skin and resistance offered by bone to electrical shock generates high-temperature gradients and is one of the contributing factors to deep muscle necrosis. Other factors are diffuse vascular injury along with the development of compartments [5,12].

The depth of pediatric electrical burn is potentiated by the fact that they have low body fat content and their surface area to volume ratios are different than adults [13].

According to the descriptive analysis of our study, it was deduced that male children belonging to older age groups were most affected by high-voltage electrical burns. Our findings with respect to age and gender were similar to that of a study in India [13]. This is also consistent with a study conducted in Iran where 97.8% of victims of electrical burns were males [14]. The most common cause of injury was either chasing a kite on the roof with a metallic rod or accidental touch while playing outdoors in the absence of a guardian. Our findings were consistent with those reported by Tiwari and Sharma from India [15]. In contrast to the consistent results from South Asia over the past decade, Talbot et al. from Boston report a dynamic shift of results from older-aged boys playing on electrical poles to young females being more affected by electrical burns at home. This change is being attributed to raising awareness about electrical burn injuries and their associated morbidity patterns [16].

Electrical burn injuries were further classified as low voltage and high voltage with 70% being high voltage and 30% being low voltage injuries. These were similar to the findings by Celik et al from an authors’ center in Turkey, with the reported incidence of high voltage injury to be 63%[12]. The total body surface involved was mostly in the range of 10-20%, while the depth of muscle injury was invariably far greater than the surface area involved. The hypothesis that the total body surface area involved and high voltage injury is associated with recovery was nullified as no significant association was found. As the extent of the injury is reported to be always greater, as discussed earlier, 43.33% of our patients landed in amputations, where mostly upper limbs were involved and unilateral amputation was done. Celik et al. reported an amputation rate of 26% [12] while Alemayehu et al. reported that 12% of the patients had to undergo rehabilitation because of amputation [17]. A study conducted by Dash et al. reported an amputation rate of 38% [18].

For the psychosocial analysis, all the survivors who were a part of the interview study were hypothesized to be having difficulties in adjusting to the daily routine of life (1. Emotional Problem Scale, 2. Conduct Problem Scale, 3. Hyperactivity Scale, 4. Peer Problem Scale, 5. Prosocial Scale, 6. Total Difficulties Scale). Most of our patients reported difficulties in dealing with peers, and social anxiety was also quite evident in most of the patients. A similar international study by Maskell et al., which was a multicentric study in New Zealand and Australia, reported the same finding, with patients of burn scarring having low scores on HRQoL (Health-Related Quality of Life scale) and SDQ with disturbed behavioral patterns [8].

Most of our patients were labeled to be having body dysmorphic disorder and mostly they were thinking about how to avoid social gatherings mainly because of their physical appearance. These patients when interviewed told that they spent more than 4 hours every day thinking about their disfigurement and how to face peers at school and in the neighborhood. While our patients were referred for proper psychological support and therapy, the data from New Zealand and Australia by Maskell et al. suggested that the hypothesis that burn patients score low on self-esteem score was nullified as no significant association was found for them [8].

No significant association was found between Total Difficulty Score with either mode of injury, mode of recovery, amputations, or application of prosthetics. Patients even with low voltage injuries were stressed similarly and comparatively to those with high voltage injuries. Haddadin from Jordan reported that the increasing length of hospital stay and minor burns when admitted were associated with a significantly low score on the strength and difficulties index [19].

For a country like Pakistan, we are way behind The Burn Plan which was a 10-year program (2008-2017) devised by the World Health Organization. The Burn Plan consisted of seven components. The first one was advocacy for the prevention of burns by raising awareness, followed by effective and sustainable burn care policies. After the implementation of these policies, data needs to be maintained, according to which research-based faster trials of promising interventions can be carried out. The remaining three goals were prevention, provision of adequate services, and capacity building 

Needless to say, we are still at the initial step and not enough has been done because of which we are still lagging behind in every aspect of burn management. The earlier the disease is recognized as a national health problem with a major financial, psychological, and physical impact on patients and their caregivers, the sooner we will be able to counter the devastating long-term effects it leaves on the community.

Our study has certain limitations. Firstly, since most patients came to our tertiary care setup through referrals from far-flung areas, in-person follow-up could not be done owing to logistical and financial constraints. We had to resort to telephonic calls for the follow-up interviews. Secondly, due to the scarcity of child psychiatrists in our setup, timely psychological support could not be provided to most of the study participants despite an evident need.

## Conclusions

Electric burn injuries are potentially life-threatening but mostly preventable, more so in the pediatric age group. The long-term psychological stress and the varied spectrum of psychiatric disorders in such patients are profound. The prevention of burn injuries can be effectively achieved by educating parents about safety measures and improving not only the health infrastructure but also through making legislation in the power supply sector. Implementation of a dedicated national program for psychological support of burn patients should be made accessible to all burn victims. Adherence to safety regulations by the people and governing bodies with respect to the placement and maintenance of power lines cannot be over-emphasized. Strict disciplinary actions against violators might help to reduce the physical and psychological morbidity and mortality associated with electrical burns. We recommend that a liaison between plastic surgeons, pediatric surgeons, and psychiatrists must be established for the proper treatment of victims of electrical burns.
